# Intravascular Imaging-Guided Versus Angiography-Guided Percutaneous Coronary Intervention in Patients with Non-ST-Segment Elevation Myocardial Infarction in the United States: Results from Big Data Analysis

**DOI:** 10.3390/jcdd12040161

**Published:** 2025-04-17

**Authors:** Chayakrit Krittanawong, Song Peng Ang, Neil Sagar Maitra, Zhen Wang, Mahboob Alam, Hani Jneid, Samin Sharma

**Affiliations:** 1 HumanX, Delaware, DE 19958, USA; 2 Division of Internal Medicine, Rutgers Health/Community Medical Center, New Brunswick, NJ 08901, USA; 3 Division of Cardiology, Scripps Clinic, La Jolla, CA 92037, USA; 4 Robert D. and Patricia E. Kern Center for the Science of Health Care Delivery, Mayo Clinic, Rochester, MN 55905, USA; 5 Division of Health Care Policy and Research, Department of Health Sciences Research, Mayo Clinic, Rochester, MN 55905, USA; 6 The Texas Heart Institute, Baylor College of Medicine, Houston, TX 77030, USA; 7 John Sealy Distinguished Centennial Chair in Cardiology, Division of Cardiology, University of Texas Medical Branch, Houston, TX 77225, USA; 8 Cardiac Catheterization Laboratory of the Cardiovascular Institute, Mount Sinai Hospital, New York, NY 10029, USA

**Keywords:** NSTEMI, IVUS, PCI, OCT

## Abstract

Non-ST-segment elevation myocardial infarction (NSTEMI) can be managed by ischemia guide strategies or early invasive strategies. Here, we present the findings of an updated contemporary analysis regarding the use of intracoronary imaging (ICI)-guided PCI versus angiography-guided PCI and in-hospital mortality in patients with NSTEMI in the United States using the NIS database from 2016 to 2021. ICI use increased by nearly threefold between 2016 and 2021, without a significant difference in in-hospital mortality, though interestingly, mortality rates compared with angiography guidance were similar and relatively low. In this study, the use of ICI was associated with lower adjusted odds of in-hospital mortality, cardiogenic shock, and cardiac arrest, but with a longer length of stay and cost of hospitalization.

## 1. Introduction

Non-ST-segment elevation myocardial infarction (NSTEMI), characterized by myocardial necrosis with biomarker elevation but lacking persistent ST-segment elevation, represents an important subset of acute coronary syndromes. While most NSTEMIs arise from type 1 myocardial infarction (MI) characterized by plaque rupture or erosion, up to 35% result from type 2 MI where there is a supply–demand mismatch. Current guidelines advocate for early invasive strategies for high-risk patients, in addition to guideline-directed medical therapy that includes dual antiplatelet therapy and anticoagulation [1].

The use of intracoronary imaging (ICI), including intravascular ultrasound (IVUS) and optical coherence tomography (OCT), has gained popularity during percutaneous coronary intervention (PCI) by allowing precise lesion assessment and stent optimization [2,3,4]. While there is evidence supporting the use of ICI in STEMI cases, its role in NSTEMI remains underexplored [5]. A study from the British Cardiovascular Intervention Society (BCIS) Registry revealed that while ICI use has increased over the last decade, the utilization rate remained low overall and was lowest in acute coronary syndrome [6].

Given the gap in the literature, we aimed to examine the real-world pattern of ICI utilization and its associated outcomes as well as resource utilization among NSTEMI patients using a national database in the United States.

## 2. Methods

### 2.1. Data Source

This study utilized the National Inpatient Sample (NIS), the largest publicly accessible all-payer inpatient database in the United States. The NIS provides reliable estimates on inpatient healthcare utilization, costs, access, quality, and outcomes across both regional and national levels. The database encompasses approximately 7 million hospital discharges annually in its unweighted form, representing an estimated 35 million hospitalizations nationwide when discharge weights are applied. Due to the anonymized nature of the NIS data, this study was exempted from Institutional Review Board approval.

### 2.2. Study Population

Hospitalizations from 2016 to 2021 were identified within the NIS database using ICD-10-CM codes (Supplementary Table S1) to capture cases of NSTEMI and PCI. Hospitalizations were categorized into angiography-guided PCI and ICI-guided PCI. ICI-guided PCI was defined in our study as individuals that underwent either IVUS or OCT-guided PCI. Exclusion criteria included patients younger than 18 years and those with missing data on age, gender, race, mortality, or household income.

### 2.3. Study Outcomes and Definitions

The primary endpoint was all-cause in-hospital mortality. Secondary outcomes included acute kidney injury, cardiogenic shock, cardiac arrest, and healthcare resource utilization metrics, such as hospital length of stay and cost. Additionally, the study assessed trends in the use of ICI among hospitalized NSTEMI patients undergoing PCI.

### 2.4. Statistical Analysis

Discharge weights provided by HCUP were applied to derive nationally representative estimates. Categorical variables were summarized as frequencies and percentages, while continuous variables were presented as means with standard deviations. Between-group comparisons were conducted using chi-square tests for categorical variables and linear regression for continuous variables. Statistical significance was defined as a two-tailed *p*-value < 0.05. Consistent with HCUP data use guidelines, cells with ≤10 observations were not reported. Temporal trends in PCI strategies and in-hospital mortality were visually represented and statistically analyzed using the Cochran–Armitage test. Outcomes for ICI-guided PCI were compared to angiography-guided PCI. Subsequently, predictors of in-hospital mortality among individuals undergoing ICI-guided PCI were analyzed. Multivariable logistic regression models, including covariates with a threshold *p*-value < 0.20, were utilized to adjust for baseline differences. To reduce confounding, we performed 1:1 propensity score matching (PSM) between ICI- and angiography-guided PCI cohorts. Propensity scores were derived from a logistic regression model incorporating 32 clinically relevant covariates, including demographics, comorbidities (e.g., diabetes, chronic kidney disease), available procedural details (use of fractional flow reserve, chronic total occlusion, type of stents, mechanical circulatory support), and hospital characteristics. Nearest-neighbor matching with a caliper width of 0.01 standard deviations achieved balanced cohorts (standardized mean differences within 0.1 post-matching). Outcomes were then compared using conditional logistic regression adjusted for HCUP sampling strata. All statistical analyses were performed using STATA version 17.0 (StataCorp, College Station, TX, USA).

## 3. Results

**Figure 1:**
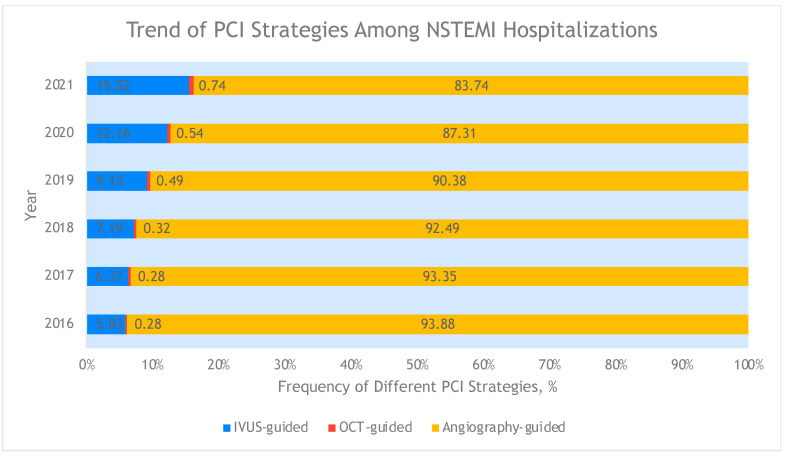
Trend in PCI strategies among NSTEMI hospitalizations. PCI: percutaneous coronary intervention; NSTEMI: non-ST elevation myocardial infarction.

**Figure 2:**
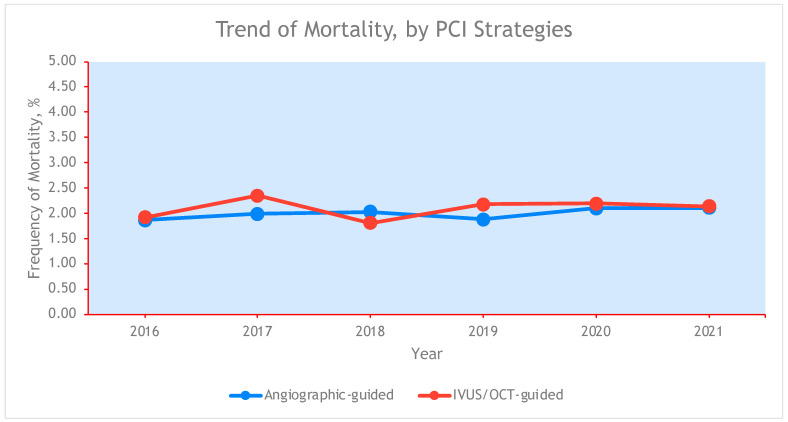
Trend in mortality by PCI strategy. PCI: percutaneous coronary intervention.

Within the 2016 to 2021 NIS database, there were data for 1,091,000 hospitalizations for NSTEMI, of which 90.3% (n = 984,940) utilized angiography-guided PCI and 9.7% (n = 106,060) utilized ICI-guided PCI. Trends in the use of ICI from 2016 to 2021 are shown in Figure 1, with a progressive yearly increase in ICI utilization from 5.8% in 2016 to 15.5% in 2021. The trends in in-hospital mortality by PCI strategy were similar from 2016 to 2021 regardless of angiography guidance or ICI guidance, as seen in Figure 2.

The baseline demographic and hospitalization characteristics are shown in Table 1. ICI was significantly less commonly utilized in Black patients (9.7 vs. 10.5%, *p* < 0.001), in small and medium-sized hospitals, in rural and urban non-teaching hospitals, in self-pay patients, in patients with a lower median household income, and in hospitals in the south. However, ICI was significantly more commonly utilized in patients with congestive heart failure, valvular heart disease, peripheral vascular disease, hyperlipidemia, liver disease, anemia, obesity, prior MI, and an Elixhauser comorbidity score > 4. ICI was also more commonly utilized in NSTEMI hospitalizations utilizing FFR, where DES was ultimately implanted, or with the use of any MCS and specifically LVAD and IABP. Similarly, predictors of ICI versus angiography guidance are shown in Table 2. Asians were less likely to have ICI (aOR 0.89 [0.80–0.99, *p* = 0.027]), while Native Americans were more likely (aOR 1.44 [1.19–1.75, *p* < 0.001]). Hospital size was predictive of ICI, with large hospitals significantly more likely to use ICI compared to small hospitals (aOR 1.33 [1.2–1.48, *p* < 0.001]). Urban teaching hospitals were more likely to use ICI compared to rural hospitals (aOR 1.21 [1.02–1.44, *p* = 0.034]). Hospitals in the west were more likely (aOR 1.42 [1.25–1.61, *p* < 0.001]) to use ICI and hospitals in the south were less likely (aOR 0.86 [0.76–0.98, *p* = 0.02]). Patients with no charge were less likely to have ICI (aOR 0.69 [0.51–0.93, *p* = 0.017]) and patients with higher median household income were more likely (aOR 1.27 [1.17–1.36, *p* < 0.001]). The presence of CHF (aOR 1.11 [1.07–1.16, *p* < 0.001]), valvular heart disease (aOR 1.10 [1.05–1.15, *p* < 0.001]), anemia (aOR 1.14 [1.06–1.23, *p* = 0.001]), hyperlipidemia (aOR 1.12 [1.07–1.16, *p* < 0.001]), CKD (aOR 1.05 [1.01–1.09, *p* = 0.02]), obesity (aOR 1.06 [1.02–1.10, *p* = 0.002]), prior MI (aOR 1.06 [1.02–1.10, *p* = 0.004]), and FFR utilization (aOR 1.85 [1.73–1.98, *p* < 0.001]) and the use of MCS (aOR 2.11 [1.97–2.25, *p* < 0.001]) were all predictors of ICI utilization.

The results of the analyses of the primary and secondary outcomes with ICI versus angiography guidance are shown in Table 3. The crude in-hospital mortality rate was higher in ICI-guided PCI compared to angiography-guided PCI (2.12% vs. 1.99%), though this difference is not statistically significant. Adjusted analyses revealed a 25% relative reduction in mortality (aOR 0.75, 95% CI 0.67–0.83; *p* < 0.001), persisting after propensity score matching (15% reduction, OR 0.85, 95% CI 0.74–0.96; *p* = 0.012). Similarly, IVUS/OCT guidance was associated with a 16% lower risk of cardiogenic shock (aOR 0.84, 95% CI 0.77–0.92; *p* < 0.001) and a 9% matched reduction (OR 0.91, 95% CI 0.83–0.99; *p* = 0.04). While unadjusted rates suggested higher acute kidney injury (AKI) with IVUS/OCT (18.89% vs. 17.36%; *p* < 0.001), this difference attenuated after multivariate adjustment (aOR 0.97, 95% CI 0.93–1.01; *p* = 0.193). Notably, IVUS/OCT-guided PCI showed a 12% lower adjusted risk of cardiac arrest (aOR 0.88, 95% CI 0.79–0.98; *p* = 0.018), though this association lost significance in propensity-matched cohorts (OR 0.90, 95% CI 0.79–1.04; *p* = 0.157). Length of stay was prolonged with ICI (4.47 ± 5.18) compared to angiography guidance (3.98 ± 4.93; *p* < 0.001). The cost of the hospitalization was increased with ICI (USD 33,177 ± 25,533) compared to angiography guidance (25,942 ± 20,647), *p* < 0.001.

## 4. Discussion

Herein, we report one of the largest, real-world analyses of trends and clinical outcomes with ICI-guided PCI compared to angiography-guided PCI for revascularization during NSTEMI. ICI use increased nearly threefold between 2016 and 2021, without a significant difference in in-hospital mortality, though interestingly, mortality rates compared with angiography guidance were similar and relatively low. In this study, the use of ICI was associated with lower adjusted odds of in-hospital mortality, as well as odds of cardiogenic shock and cardiac arrest, with longer length of stay and higher cost of hospitalization. As expected, large urban teaching hospitals had increased utilization of ICI. This could perhaps be due to the patient population being much sicker.

Contemporary studies of ICI for NSTEMI have demonstrated improved outcomes when compared to angiography guidance alone, including for all-cause mortality, MACE, and target vessel revascularization [2], and reduced MACE is even seen beyond one year after PCI when IVUS guidance is utilized [7] and even up to three years after [8]. Multinational, randomized data show lower composite cardiac death, target vessel MI, or clinically driven revascularization rates with IVUS use [9]. Specifically, patients with complex lesions benefit the most in terms of MACE [10]. However, performing complex PCI in NSTEMI patients is associated with a significantly increased risk of periprocedural myocardial infarction, which substantially raises short- and long-term mortality risk in this population, as recently demonstrated for the first time [11]. ICI allows for the optimization of PCI through appropriate stent sizing/selection and deployment, maximizing the minimal stent area (MSA), and precision placement to avoid edge disease or dissection or to recognize and correct complications at the time of the index PCI. Our study was largely in line with previous studies. Although the crude rate of in-hospital mortality was numerically higher in ICI-guided PCI, the adjusted odds of in-hospital mortality were significantly lower with ICI-guided PCI. We deduced that this is related to increased ICI use in complex PCI cases, which are more typically attributed a higher procedural risk. Therefore, after propensity score matching accounting for individual demographics, comorbidities, and hospital-level variables, the mortality rate was significantly reduced with the use of ICI. Indeed, sicker patients beget longer, more costly hospitalizations, as seen in our study.

The temporal trend of increasing ICI use in US hospitalizations is reassuring, particularly as it is being utilized in sicker, complex cardiac patients in the hope of improving outcomes, as seen in contemporary data. Prior studies have shown ICI use, namely IVUS, to be as low as 5% based on data from 2009 to 2017 [12]. In our study, the increased use is seen primarily in large urban teaching hospitals in the west. Further research should evaluate barriers to ICI adoption, which may include productivity-based pressures, resource-based limitations, and/or simply clinical inertia.

The NIS database is a large, robust dataset containing real-world information from all US hospitalizations, rather than research-based registries or trials only. As such, our temporal trends and predictors of utilization data are informative of overall clinical practice. However, several limitations remain. Retrospective analysis of the NIS database provides limited insight into ACS-specific data such as specific lesion characteristics, angiographic data, PCI complication information, and number of stents used. Furthermore, the NIS does not include data on whether specific ICI protocols (e.g., systematic evaluation of stent expansion, apposition, or malposition) were employed during PCI. This precludes the assessment of how protocol-driven ICI utilization influences clinical outcomes, despite guidelines emphasizing its importance for stent optimization. The identification of cases is based on billing codes, which raises the possibility of selection bias or errant inclusion in situations such as type II NSTEMI that ultimately underwent angiography, which represents a different clinical entity than true ACS and can confound findings. Lastly, the NIS dataset includes individual hospitalization data per annum and may not capture information regarding complications such as target vessel MI or revascularization, nor long-term and many short-term outcomes.

## 5. Conclusions

We present a large-scale, real-world study of ICI use for NSTEMI patients, demonstrating threefold increased utilization of ICI compared to angiography alone and increased utilization in large urban teaching hospitals in the Western US from 2016 to 2021. ICI-guided PCI was associated with lower adjusted odds of in-hospital mortality compared to angiography-guided PCI. Further longitudinal studies are warranted to validate these findings.

## Figures and Tables

**Table 1:** Baseline characteristics comparison between intracoronary imaging-guided and angiography-guided percutaneous coronary intervention (PCI).

Variables	Angiography-Guided (n = 984,940)	ICI-Guided (n = 106,060)	Total (n = 1,091,000)	*p*
**Age**	66.23 ± 12.46	66.39 ± 12.50	66.24 ± 12.46	0.102
**Female**	343,170 (34.84)	36,345 (34.27)	379,515 (34.79)	0.1065
**Race**				<0.001
White	741,560 (75.29)	79,100 (74.58)	820,660 (75.22)	
Black	103,045 (10.46)	10,310 (9.72)	113,355 (10.39)	
Hispanic	80,410 (8.16)	8850 (8.34)	89,260 (8.18)	
Asian or Pacific Islander	25,375 (2.58)	3205 (3.02)	28,580 (2.62)	
Native American	5685 (0.58)	930 (0.88)	6615 (0.61)	
Other	28,865 (2.93)	3665 (3.46)	32,530 (2.98)	
**Hospital Bed Size**				<0.001
Small	162,335 (16.48)	14,905 (14.05)	177,240 (16.25)	
Medium	299,675 (30.43)	26,990 (25.45)	326,665 (29.94)	
Large	522,930 (53.09)	64,165 (60.50)	587,095 (53.81)	
**Hospital Teaching Status**				<0.001
Rural	59,665 (6.06)	5435 (5.12)	65,100 (5.97)	
Urban Non-teaching	202,555 (20.57)	18,620 (17.56)	221,175 (20.27)	
Urban Teaching	722,720 (73.38)	82,005 (77.32)	804,725 (73.76)	
**Admission**				0.7649
Elective	46,925 (4.76)	5115 (4.82)	52,040 (4.77)	
**Primary payment coverage**				<0.001
Medicare	557,375 (56.59)	60,810 (57.34)	618,185 (56.66)	
Medicaid	87,605 (8.89)	9485 (8.94)	97,090 (8.90)	
Private insurance	264,490 (26.85)	28,425 (26.80)	292,915 (26.85)	
Self-pay	42,670 (4.33)	3925 (3.70)	46,595 (4.27)	
No charge	4185 (0.42)	265 (0.25)	4450 (0.41)	
Other	28,615 (2.91)	3150 (2.97)	31,765 (2.91)	
**Median household income, USD**				<0.001
1–28,999	303,835 (30.85)	28,860 (27.21)	332,695 (30.49)	
29,000–35,999	273,740 (27.79)	27,025 (25.48)	300,765 (27.57)	
36,000–46,999	232,390 (23.59)	26,530 (25.01)	258,920 (23.73)	
47,000+	174,975 (17.77)	23,645 (22.29)	198,620 (18.21)	
**Hospital Region**				<0.001
Northeast	157,880 (16.03)	17,810 (16.79)	175,690 (16.10)	
Midwest	234,935 (23.85)	24,980 (23.55)	259,915 (23.82)	
South	422,420 (42.89)	36,435 (34.35)	458,855 (42.06)	
West	169,705 (17.23)	26,835 (25.30)	196,540 (18.01)	
**Comorbidities**				
Congestive heart failure	377,540 (38.33)	45,240 (42.66)	422,780 (38.75)	<0.001
Atrial fibrillation	145,165 (14.74)	15,580 (14.69)	160,745 (14.73)	0.8539
Valvular heart diseases	130,020 (13.20)	16,215 (15.29)	146,235 (13.40)	<0.001
Peripheral vascular disease	120,515 (12.24)	13,720 (12.94)	134,235 (12.30)	0.0036
Hypertension	833,140 (84.59)	89,550 (84.43)	922,690 (84.57)	0.5922
Chronic lung disease	210,935 (21.42)	22,675 (21.38)	233,610 (21.41)	0.9047
Diabetes	436,820 (44.35)	46,690 (44.02)	483,510 (44.32)	0.3861
Hyperlipidemia	730,170 (74.13)	80,410 (75.82)	810,580 (74.30)	<0.001
CKD	237,855 (24.15)	27,920 (26.32)	265,775 (24.36)	<0.001
Liver disease	30,840 (3.13)	3705 (3.49)	34,545 (3.17)	0.004
Anemia	33,020 (3.35)	4315 (4.07)	37,335 (3.42)	<0.001
Cancer	24,315 (2.47)	2845 (2.68)	27,160 (2.49)	0.0525
Obesity	227,690 (23.12)	25,685 (24.22)	253,375 (23.22)	0.0012
Alcohol use	29,320 (2.98)	2895 (2.73)	32,215 (2.95)	0.0507
Smoking	240,780 (24.45)	24,120 (22.74)	264,900 (24.28)	<0.001
Prior MI	179,280 (18.20)	20,330 (19.17)	199,610 (18.30)	0.0013
Prior PCI	14,395 (1.46)	1150 (1.08)	15,545 (1.42)	<0.001
Prior CABG	112,060 (11.38)	8795 (8.29)	120,855 (11.08)	<0.001
Elixhauser comorbidity score ≥ 4	486,435 (49.38)	55,945 (52.78)	542,380 (49.71)	<0.001
**Angiographic characteristics**				
CTO	45,565 (4.63)	5060 (4.77)	50,625 (4.64)	0.3855
FFR	40,060 (4.07)	7945 (7.49)	48,005 (4.40)	<0.001
DES	882,740 (89.62)	98,180 (92.57)	980,920 (89.91)	<0.001
BMS	53,080 (5.39)	3585 (3.38)	56,665 (5.19)	<0.001
**In-hospital management**				
Use of MCS	35,320 (3.59)	8340 (7.86)	43,660 (4.00)	<0.001
LVAD	19,095 (1.94)	5620 (5.30)	24,715 (2.27)	<0.001
ECMO	770 (0.08)	125 (0.12)	895 (0.08)	0.0546
IABP	17,755 (1.80)	3305 (3.12)	21,060 (1.93)	<0.001

PCI: percutaneous coronary intervention; MI: myocardial infarction; CABG: Coronary Artery Bypass Grafting; FFR: fractional flow reserve; CTO: chronic total occlusion; BMS: bare-metal stent; DES: drug-eluting stent; MCS: mechanical circulatory support; CKD: chronic kidney disease; LVAD: Left Ventricular Assist Device; ECMO: Extracorporeal Membrane Oxygenation; IABP: Intra-Aortic Balloon Pump.

**Table 2:** Predictors of patients receiving intracoronary imaging (ICI).

Variables	aOR (95% CI)	*p*
**Age**		
Age < 65	Ref	
Age ≥ 65	0.97 (0.92–1.01)	0.157
**Sex**		
Male	Ref	
Female	0.97 (0.94–1.00)	0.096
**Race**		
White	Ref	
Black	1.01 (0.94–1.09)	0.705
Hispanic	0.97 (0.90–1.05)	0.442
Asian or Pacific Islander	0.89 (0.80–0.99)	0.027
Native American	1.44 (1.19–1.75)	<0.001
Other	1.11 (1.00–1.25)	0.06
**Hospital Bed Size**		
Small	Ref	
Medium	0.99 (0.88–1.10)	0.815
Large	1.33 (1.20–1.48)	<0.001
**Hospital Teaching Status**		
Rural	Ref	
Urban Non-teaching	0.98 (0.81–1.17)	0.795
Urban Teaching	1.21 (1.02–1.44)	0.034
**Hospital Region**		
Northeast	Ref	
Midwest	0.99 (0.87–1.13)	0.882
South	0.86 (0.76–0.98)	0.02
West	1.42 (1.25–1.61)	<0.001
**Primary payment coverage**		
Medicare	Ref	
Medicaid	0.96 (0.90–1.02)	0.202
Private insurance	0.99 (0.94–1.04)	0.773
Self-pay	0.97 (0.88–1.06)	0.479
No charge	0.69 (0.51–0.93)	0.017
Other	1.03 (0.94–1.13)	0.552
**Median household income, USD**		
1–28,999	Ref	
29,000–35,999	1.01 (0.96–1.06)	0.789
36,000–46,999	1.11 (1.04–1.17)	0.001
47,000+	1.27 (1.17–1.36)	<0.001
Congestive heart failure	1.11 (1.07–1.16)	<0.001
Valvular heart disease	1.10 (1.05–1.15)	<0.001
Peripheral vascular diseases	1.01 (0.96–1.06)	0.703
Atrial fibrillation	0.93 (0.89–0.97)	0.001
Hypertension	0.96 (0.91–1.00)	0.052
Anemia	1.14 (1.06–1.23)	0.001
Cancer	1.06 (0.97–1.15)	0.223
Diabetes	0.94 (0.91–0.97)	<0.001
Hyperlipidemia	1.12 (1.07–1.16)	<0.001
Smoking	0.98 (0.95–1.02)	0.366
CKD	1.05 (1.01–1.09)	0.02
Chronic liver disease	0.96 (0.89–1.04)	0.376
Obesity	1.06 (1.02–1.10)	0.002
Alcohol use	0.91 (0.83–1.00)	0.044
Prior PCI	0.76 (0.66–0.88)	<0.001
Prior MI	1.06 (1.02–1.10)	0.004
Prior CABG	0.71 (0.67–0.75)	<0.001
FFR	1.85 (1.73–1.98)	<0.001
CTO	1.02 (0.95–1.10)	0.55
BMS	0.77 (0.69–0.86)	<0.001
DES	1.24 (1.16–1.33)	<0.001
Use of MCS	2.11 (1.97–2.25)	<0.001
Elixhauser score ≥ 4	1.05 (1.00–1.10)	0.037

aOR: Adjusted Odds Ratio; CI: Confidence Interval; PCI: percutaneous coronary intervention; MI: Myocardial Infarction; CABG: Coronary Artery Bypass Grafting; FFR: fractional flow reserve; CTO: chronic total occlusion; BMS: bare-metal stent; DES: drug-eluting stent; MCS: mechanical circulatory support; CKD: chronic kidney disease.

**Table 3:** Comparison of outcomes between intracoronary imaging (ICI) and angiography-guided percutaneous coronary intervention (PCI).

In-Hospital Outcomes	Angiography-Guided (n = 984,940)	ICI-Guided (n = 106,060)	Total (n = 1,091,000)	*p*	aOR (95% CI)	*p*	PSM OR (95% CI)	*p*
Mortality	19,620 (1.99)	2245 (2.12)	21,865 (2.00)	0.222	0.75 (0.67–0.83)	<0.001	0.85 (0.74–0.96)	0.012
AKI	171,005 (17.36)	20,040 (18.89)	191,045 (17.51)	<0.001	0.97 (0.93–1.01)	0.193	1.00 (0.95–1.06)	0.874
Cardiogenic shock	33,485 (3.40)	4890 (4.61)	38,375 (3.52)	<0.001	0.84 (0.77–0.92)	<0.001	0.91 (0.83–0.99)	0.04
Cardiac Arrest	18,120 (1.84)	2010 (1.90)	20,130 (1.85)	0.5694	0.88 (0.79–0.98)	0.018	0.90 (0.79–1.04)	0.157
Length of stay	3.98 ± 4.93	4.47 ± 5.18	4.03 ± 4.96	<0.001	-	-		
Cost of hospitalization	25,942 ± 20,647	33,177 ± 25,533	26,642 ± 21,277	<0.001	-	-		

Angiography-guided as reference group. AKI: acute kidney injury; aOR: Adjusted Odds Ratio; CI: Confidence Interval; PCI: percutaneous coronary intervention; PSM: propensity-score matching.

## Data Availability

The original contributions presented in this study are included in the article. Further inquiries can be directed to the corresponding author.

## Supplementary Materials

The following supporting information can be downloaded at: https://www.mdpi.com/article/10.3390/jcdd12040161/s1, Table S1: ICD-10 codes.

## Abbreviations

aOR: Adjusted Odds Ratio; CI: Confidence Interval; PCI: percutaneous coronary intervention; MI: Myocardial Infarction; CABG: Coronary Artery Bypass Grafting; FFR: fractional flow reserve; CTO: chronic total occlusion; BMS: bare-metal stent; DES: drug-eluting stent; MCS: mechanical circulatory support; CKD: chronic kidney disease; LVAD: Left Ventricular Assist Device; ECMO: Extracorporeal Membrane Oxygenation; IABP: Intra-Aortic Balloon Pump; PSM: propensity-score matching.
