# Supplementary material for: Intravascular Imaging-Guided Versus Angiography-Guided Percutaneous Coronary Intervention in Patients with Non-ST-Segment Elevation Myocardial Infarction in the United States: Results from Big Data Analysis
Source: J Cardiovasc Dev Dis. 2025 Apr 17;12(4):161. doi: 10.3390/jcdd12040161 (PMC12027885; doi:10.3390/jcdd12040161)
Supplement: Supplementary file 1 [file jcdd-12-00161-s001.zip › jcdd-3482445-supplementary.pdf]

Supplementary Material

Table S1. ICD-10 codes.

Variables	ICD-10 codes
NSTEMI	I21.4
PCI	0270346 027034Z 0270356 027035Z 0270366 027036Z 0270376 027037Z 02703D6 02703DZ 02703E6 02703EZ 02703F6 02703FZ 02703G6 02703GZ 02703Z6 02703ZZ 0270446 027044Z 0270456 027045Z 0270466 027046Z 0270476 027047Z 02704D6 02704DZ 02704E6 02704EZ 02704F6 02704FZ 02704G6 02704GZ 02704Z6 02704ZZ 0271346 027134Z 0271356 027135Z 0271366 027136Z 0271376 027137Z 02713D6 02713DZ 02713E6 02713EZ 02713F6 02713FZ 02713G6 02713GZ 02713Z6 02713ZZ 0271446 027144Z 0271456 027145Z 0271466 027146Z 0271476 027147Z 02714D6 02714DZ 02714E6 02714EZ 02714F6 02714FZ 02714G6 02714GZ 02714Z6 02714ZZ 0272346 027234Z 0272356 027235Z 0272366 027236Z 0272376 027237Z 02723D6 02723DZ 02723E6 02723EZ 02723F6 02723FZ 02723G6 02723GZ 02723Z6 02723ZZ 0272446 027244Z 0272456 027245Z 027246Z 0272476 027247Z 02724D6 02724DZ 02724E6 02724EZ 02724F6 02724FZ 02724G6 02724GZ 02724Z6 02724ZZ 0273346 027334Z 0273356 027335Z 0273366 027336Z 0273376 027337Z 02733D6 02733DZ 02733E6 02733EZ 02733F6 02733FZ 02733G6 02733GZ 02733Z6 02733ZZ 0273446 027344Z 0273456 027345Z 0273466 027346Z 0273476 027347Z 02734D6 02734DZ 02734E6 02734EZ 02734F6 02734G6 02734GZ 02734Z6 02734ZZ
OCT	B221Z2Z, B223Z2Z
IVUS	B241ZZ3 B240ZZ3
Cardiogenic shock	R57
Acute kidney injury	N17x, N990
